# Reconsidering Empathy: An Interpersonal Approach and Participatory Arts in the Medical Humanities

**DOI:** 10.1007/s10912-021-09701-6

**Published:** 2021-06-08

**Authors:** Erica L. Cao, Craig D. Blinderman, Ian Cross

**Affiliations:** 1 University of Cambridge, Cambridge, UK; 2 Columbia University College of Physicians and Surgeons, New York City, NY USA; 3 Columbia University Irving Medical Center, New York City, NY USA

**Keywords:** Medical humanities, Music, Medical education, Arts and health, Narrative medicine

## Abstract

The decline of empathy among health professional students, highlighted in the literature on health education, is a concern for medical educators. The evidence suggests that empathy decline is likely to stem more from structural problems in the healthcare system rather than from individual deficits of empathy. In this paper, we argue that a focus on direct empathy development is not effective and possibly detrimental to justice-oriented aims. Drawing on critical and narrative theory, we propose an interpersonal approach to enhance empathic capacities that is centered on constructive and transformative interactions which integrates the participatory arts and involves both patients and health professional students. We describe and evaluate a program where patients and students create collaborative, original songs. Interviews and a focus group revealed interactional processes summarized in four themes: reciprocal relationships, interactions in the community, joint goal, and varied collaboration. There was a significant enhancement of positive attitudes about care post-program amongst health professional students. The interpersonal approach may be a preliminary framework for the medical humanities to shift away from a focus on direct empathy development and further towards participatory, co-creative, and justice-oriented approaches to enhance health and thereby empathic capabilities.

## The empathy approach

It is common to think of empathy as a skill, trait, or sometimes, in medical education, a “competency.” Among medical educators, there has been particular concern over the decline of empathy throughout clinical training—especially in the year when medical students begin clinical rotations (Chen et al. 2007). Proposed factors driving this decline include ethical erosion, loss of idealism (Vaidyanathan 2015), and a tendency in medical education to privilege a medico-scientific rather than a biocultural understanding of clinical process (Pedersen 2010). Social isolation, loss of control, and lack of meaning in work are partly influenced by inefficiency in electronic health record systems and reporting requirements under a healthcare system led, in the USA and elsewhere, by corporate and bureaucratic interests. A range of factors related to the failings of healthcare systems contribute to burnout and decline in empathic attitudes, suggesting that the kind of “empathy” in decline is socially contextualized rather than a trait characteristic.

Empathy is generally understood among psychologists as a cognitive and affective ability to understand the thoughts and feelings of others. In this sense, empathy is conceptualized as a trait characteristic or skill amenable to measurement through tools such as through the Interpersonal Reactivity Index, a questionnaire which asks respondents how they typically react in situations which elicit “empathic concern, perspective-taking, fantasy seeking, or personal distress” (Davis 1983). Other psychologists conceive of empathy as less skill or trait than as malleable and contextual. Recent work on “empathic growth” explores how social context, such as whether others are similar or dissimilar to ourselves, influence the capacity to empathize in a given situation (Weisz and Zaki 2017; Zaki 2014). Therefore while in a majority of studies empathy has been approached as a trait characteristic (Cuff et al. 2016; Davis 1983), in others, empathy is a state or attitude elicited by distal and proximal environments (Weisz and Zaki 2017; Zaki 2014).

The Jefferson Scale of Physician Empathy measures empathy as an attitude, unlike the Interpersonal Reactivity Index which measures empathy as a trait, through statements such as “I believe that empathy is an important therapeutic factor in medical treatment,” “I believe that emotion has no place in the treatment of medical illness,” and “My patients feel better when I understand their feelings” (Hojat et al. 2001). Rather than as a scale of “empathy,” some studies prefer to describe the Jefferson Scale as measuring attitudes about care or “empathic attitudes” (e.g., Crandall and Marion 2009). These attitudes are developed in students through the cultures of a clinical environment which are transmitted to physicians and then to students (e.g., see the “hidden curriculum,” Hafferty 1998).

Most studies of empathy in medical education use the Jefferson Scale which measures attitudes, yet empathy development as a trait characteristic—framed in terms of skill or competency—remains a focus in medical education and, by extension, the medical humanities (Bleakley 2015; Evans 2002; Gordon 2005; Macnaughton 2000). The skills students are expected to develop through the arts and humanities include competencies such as humanism, empathic listening, and narrative understanding. Despite the value of developing such skills in any individual, an approach centered around empathy as a skill or trait to be developed is incongruent with studies which measure empathy as attitudinal: as “I believe” statements transmitted through the values and norms of the profession.

Empathy has been critiqued in both medical education and social sciences as inappropriate for the physician stance of detachment (Macnaughton 2009) and prone to implicit bias (Bloom 2017). In contrast, our critique of empathy applies to how empathy has been approached in clinical and educational contexts, namely as, an individual approach focused on the learner rather than a reciprocal interaction reflecting a broader understanding of empathy—and its history in sympathy and social context. The approach to empathy as a primarily individual construct may detract from the very goal of enhancing empathic capacities. In the context of health care, the approach towards empathy development as a solution to address burnout or empathy decline itself can be ineffective and even detrimental to justice-oriented aims, as we shall demonstrate.

## The empathy approach reconsidered

When empathy is described as a skill or trait to enhance, the burden falls on the individual rather than on the cultures and structures in place. To develop empathy in this context is to attempt to humanize a system only around the edges and not transform it at its core. The distinction between traits and skills versus attitudes in our usage is the approach to which they are developed—the former targeting the individual development of traits and skills, the latter targeting the interpersonal and social norms that shape attitudes. The former is an *individual* empathy (how empathy has typically been defined in the medical literature and in the twentieth century), the latter is an *interpersonal* empathy (drawing on historical roots of empathy in nineteenth century sympathy) (see Garden 2007). It may be argued that interpersonal empathy is more malleable and susceptible to teaching compared to individual empathy, as individuals learn what is socially sanctionable and morally permissible through social norms akin to Adam Smith’s “sympathy”—the precursor to modern-day empathy—in his *Theory of Moral Sentiments* (1759).

Words used to describe the medical humanities like “fortifies, utilize, improve, facilitate” are often applied to empathy—in the individual sense—and appear to reflect a tendency of habituation, as noted by a teacher of narrative medicine (Filippaki 2020). Scholars like Rita Charon have argued that rather than exercises in empathy forced upon students, narrative medicine, for instance, enhances the practice of medicine and justifies the relevance of the field (Lau 2019). While laudable in aim, the medical humanities may at times fall prey to frameworks which affirm power differentials, reflective of statements such as “the state of heightened focus and commitment that a listener can donate to a teller” (Charon et al. 2016, 3) or "physicians are conspicuous members of their cultures, anointed as agents of social control who deploy special powers to rescue, heal, and take command" (Charon 2001, 1899).

Health professionals often describe themselves as trapped in a system perpetuating inequality, which, in the context of US health care, has been described as “robbing the American dream” (Brownlee 2017). The root problems are not individual deficits of trait empathy but structural forces that hinder the provision of high quality and affordable care; public health researchers are calling on physicians to play a necessary role in addressing social determinants of health (Maani and Galea 2020; Metzl and Hansen 2014). Medical humanities, including narrative medicine, recognize and integrate structural and justice-oriented approaches in their work. We propose an approach to further such work. Rather than dichotomies of listener and teller, the arts can expand frameworks so that all are equal creators, or as in Augusto Boal’s *Theatre of the Oppressed,* “spectactors” (1974). Through co-creation in the participatory arts such as music-making and theater, we propose a justice-oriented interpersonal approach for the medical humanities.

## An interpersonal approach

Critical theorist Nancy Fraser (1995) describes remedies or interventions as “affirmative” when they address inequalities or imbalances without disrupting the underlying frameworks which give rise to them; they are “transformative” when they correct inequalities by transforming the framework. The former tends to promote group differentiation while the latter tends to blur it. The empathy approach can be interpreted as “affirmative”—heightening boundaries through empathic “powers” which affirm underlying structures. Our question is how to implement a boundary-blurring, transformative role for the arts and humanities in the clinical and educational context.

One reason that such an aim has been difficult to realize in the medical humanities is because what has been regarded as appropriate engagement with the arts has tended to take the form of appreciation rather than participation. The nineteenth-century idea of art as primarily valuable because of its aesthetic qualities sets it in a presentational context in which there is a clear differentiation between the “expert” producer of art and the “inexpert” consumer who has agency limited only to responding rather than initiating. Such presentational arts contrast with participatory arts such as group music-making often found in indigenous cultures or group chorus in religious contexts (Turino 2008). Most examples of the arts in medical humanities application engage in Western presentational uses: narrative workshops where students read and reflect upon literature (e.g., Charon 2001); visual arts where students go to art museums (e.g., Gowda et al. 2018), and, in the only case of music incorporation we could find, music listening in a classroom space (Newell and Hanes 2003). These approaches are primarily presentational in listening or appreciation rather than participatory in co-creation. Hence the use of the presentational type of engagement with the arts in medical education can be viewed as exemplifying the very kind of affirmative remedy that Fraser critiques; namely that, they center around cultural respect or “recognition” yet perpetuate power imbalances which do not further social equality or “redistribution.” Applying this view through a Western nineteenth-century lens to music imposes (largely unacknowledged) ethnocentric constraints on ways in which we can conceive of engagement with music and its potential effects. Following a distinction drawn by the ethnomusicologist Thomas Turino (2008), music that is “participatory,” where anyone can join in, can have direct and immediate socio-functional value (see, e.g., Rabinowitch et al, 2013; Fancourt et al, 2016; Fancourt, 2017), which does not seem to be shared by music that is primarily “presentational” such as western classical concert music.

Although the predominantly presentational arts in medical humanities allow students and teachers to engage in reflection, build relationships, and encourage aesthetic appreciation, the incorporation of participatory art, such as group music-making or collaborative songwriting, expands ways for the medical humanities to expand towards justice-oriented methods and aims through an interpersonal approach: to find an appropriate balance between recognition and redistribution so that all involved—especially if also involving patients—may jointly construct relations and realities. This integration can be seen as grounded in narrative theory after Bruner (1991). Narrative organizes human memories and experiences by "constructing and representing the rich and messy domain of human interaction," (4) not simply representing reality but constructing reality: “The daunting task that remains now is to show in detail how, in particular instances, narrative organizes the structure of human experience—how, in a word, ‘life’ comes to imitate ‘art’ and vice versa” (21). This conceptualization challenges the framework of “powers,” for powers do not just reside within an individual but are "supported and organized by cultural tool kits"—which, especially in hierarchical clinical settings, can be co-constructive through the participatory arts.

## Interpersonal approach applied: music program and evaluation

What makes music so amenable to being “participatory,” so that we often can’t help joining in? As an art form, it is uniquely multisensory, temporal, and ambiguous—it exists in time and rhythm, engages senses and affect, requires neither words nor semantic meaning. Music has “floating intentionality” in that its meanings need not stay constant between contexts, yet it incorporates temporal frameworks that allow time to be shared and that endow it with properties of an "honest signal" so all participants can feel as though *their* feelings are shared by all (Cross 1999, 2005). In other words, there is no necessity for individuals to align understandings or interpretations in order to be in sync, in rhythm and affiliation, and, unlike interaction in speech, there is no requirement on participants to make their interpretations mutually explicit for each other (Cross, 2014). When combined with narrative through songwriting, music offers further opportunities to construct non-conflictual "life stories" (after Bruner). Accordingly, we designed and evaluated a collaborative songwriting program as an example of a participatory art intervention grounded in an interpersonal approach.

Ten student participants and ten patient participants volunteered to participate in June–August 2017. Student participants were rising second- and third-year students from Columbia’s Dental, Nursing, and Medical Schools. Patient participants were members of pancreatic and breast cancer support groups led by a social worker and organized through C.B. (Director of Palliative Care) at Columbia University Irving Medical Center/New York-Presbyterian Hospital. The program consisted of 1) orientation and interest session (with students); 2) support group session #1 and conversation (with students and patients); 3) song development and recording (with students); and 4) support group session #2 and song sharing (with students and patients).

### Orientation session

Information about an orientation session was emailed to students through the health professional schools’ student clubs listserv with a summary of the project and that no musical experience was needed to participate. The one-hour session was led by E.C. with student leaders of the school’s music society. The session outlined the process and timeline of the program as well as an introduction to songwriting for those with no prior experience. Students were also given a step-by-step guide to songwriting handout and video references (see https://humansinharmony.org/engagelearn/ for songwriting resources).

### Support group session #1 and conversation

We partnered with existing breast and pancreatic cancer support group sessions held monthly at the hospital affiliated with the health professional school. The program was introduced to the patients in the groups through social worker, and patients participated on a voluntary basis. In the first session, students and patients met at the hospital conference room where support group sessions were usually held. Everyone introduced themselves and broke into pairs based on their location in the room. They then spent thirty minutes in conversation to get acquainted. To help spark conversation, each pair was provided a “song ideas form” as a guide to prompt them about the people, ideas, and places that were important to them as well as their favorite musical styles and songs (see Appendix A for song ideas form).

### Song development and recording

Due to logistical challenges of transportation, it was difficult for patients to workshop songs at the music rooms located in the student residence building. Over the course of the next four weeks, students, either with their own instruments or during optional workshops drew on the shared topics and themes of interest which emerged from the conversation at support group session #1. Oftentimes, the songs featured shared interests between patient and students, themes of family or friendship, messages of hope or strength, and preferred musical styles such as pop or jazz. In the third week (one week before the support group session), students signed up for thirty-minute slots where, with the assistance of musical classmates, they recorded the songs using a portable Zoom H4N recording device, which could be easily shared with patients when they reconvened at the next support group session.

### Support group session #2 and song sharing

With the song files downloaded onto laptops or phones, the students brought along their electronic devices and earphones to the support group session. Once again, students and patients gathered into the same pairs as in the first session. This time, scattered in various corners of the conference room, the pairs shared the songs with earphones and discussed the meanings and connections. Once each student and patient shared the song in pairs (approximately 15 min), everyone gathered as a large group. Those pairs who wished had the opportunity to share the song and meanings of the song with the larger group. As pairs volunteered to play their song through a portable speaker, they usually introduced the song and the ways the song reflected the stories and experiences shared between the pair. After each song was played, the rest of the group added comments on how the song resonated to other group members, the community of the group, and the group’s understanding of themes with the personalities they knew. The group song-sharing lasted about forty-five minutes, about seven minutes each for about six songs (not every pair chose to share their song), and then the group engaged in a focus group discussion on their experiences.

After the session’s conclusion, for those who had marked on a sharing permission form distributed at the end of the session their willingness to share the song publicly, songs were uploaded onto a SoundCloud page (e.g., https://soundcloud.com/humansinharmony/sets/survivors) so that participants could easily share with family and friends. Song recordings were also sent by email to the social worker who sent them to patients and to students for everyone to keep.

## Evaluation

In this program of collaborative songwriting, a participatory art form, what forms of interaction are engaged in? What changes, if any, in interpersonal measures such as attitudes about care occur? For the first question, we conducted interviews and a focus group to understand how patients and students interacted with each other. For the second question, we administered a questionnaire of empathic attitudes designed for health professional students to student participants; we predicted that interpersonal qualities, like attitude, would increase after participation in the program.

### Procedure and measures

Patients and students gave consent to participate in the study which was approved by the Columbia University Medical Center IRB (Protocol IRB-AAAR5141).

*Patient and public involvement.* Student representatives and the social worker leading the support groups were involved in designing the evaluation process and advised on appropriate length and applicability of questionnaires and focus group topics.

1. What kinds of meaning, engagement, and interaction occurred between patients and student participants?
  *Focus group with patients and students.* Student and patient participants, as well as social workers leading the support group, engaged in a focus group at the end of the second session. The discussion naturally took its own course in sharing participant interactions and experiences without much prompting. The focus group was audio-recorded and later transcribed. Student representatives and the social worker leading support groups were involved in designing the evaluation process and advised on appropriate length and applicability of questionnaires and focus group topics.
  Student participants (N = 10) also completed one-on-one interviews with researcher E.C. in a private recording room. The interviews took place before the second session after each student recorded their song (where students were already in a private space) and each interview lasted approximately fifteen minutes. An interview guide (see Appendix B) developed by E.C. explored how participants engaged in the process of writing and sharing songs.
  We used a general inductive qualitative approach to analyze the interviews and focus group. E.C. and colleagues A.B. and S.D. analyzed interview responses to code themes informed by the intent to further understand what kinds of interactive processes participants engaged in and found meaningful in the collaborative songwriting activity. Codes were managed on Dedoose (V. ***8.0.35***, SocioCultural Research Consultants, Los Angeles, CA).
2. What, if any, was the extent of attitude change in student participants?
  *Student questionnaires.* Student participants completed questionnaires at the beginning of the first session and at the end of the second session. Seven out of the ten participants were present for both sessions, completing both the pre- and post-program questionnaires (four women and three men ranging in age from twenty-three to twenty-eight). Not all participants were consistent in attending each session because of scheduling conflicts and the voluntary nature of the sessions as a student-run extracurricular activity. If participants couldn’t attend sessions in person, they recruited friends to participate, so that every song was completed and shared. Participants were recruited without explicit regard to musical experience. Four had never written a song before, and three had written songs before. They had an average of 14.6 years of prior musical experience.
  Students were asked to complete the Jefferson Scale of Physician Empathy (Hojat et al. 2001) at the beginning of the first session and at the end of the second session. The Jefferson Scale was the main measurement of interest because we were interested in interpersonal measurements like attitudes. Statements in the Jefferson Scale generally focus on belief about the importance of emotion in patient care, and thus measures responses shaped by norms and expectations in a particular setting. The scale has twenty statements on attitudes about care such as “An important component of the relationship with my patients is the understanding of the emotional status of themselves and their families,” and “I believe that emotion has no role in the treatment of medical illness.” We were also interested in whether trait empathy was affected, and therefore also included as a measurement the Interpersonal Reactivity Index (Davis 1983): twenty-eight statements which measure empathy as a trait characteristic which include: “I often have tender, concerned feelings for people less fortunate than me,” and “Before criticizing somebody, I try to imagine how I would feel if I were in their place.”

## Results

1. What kinds of meaning, engagement, and interaction occurred between patient and student participants?

Four themes emerged from participant responses in the interviews and focus group discussion:

- *Reciprocal relationships*. There was a dialogic process between student and patient in sharing stories, finding common ground, and learning from each other. In one pair, the student and patient connected over a shared interest in jazz and French, so that the resulting song created was in a jazzy style and one stanza was in French. One participant said, “…I have experienced where love can be so deep it can make me cry. It seems like [the patient] has this experience all the time and has had that throughout her life. I don’t think I’ve ever found someone that has brought that up before…It was something that called me…I learned a lot from her.”
- *Interactions in the community.* The support group setting allowed for vulnerability and trust to deepen and develop on a group level. One participant said, “Attending the group and seeing how they share with each other inspired me to share more in the song. They are all so open with us about who they were. It made the process more intimidating but more meaningful.” Another participant described, “Just to be a part of this, this beautiful group of people is something that no one will understand except for us, and you just made that bond stronger. It's just an incredible bond.”
- *Joint goal.* Every participant was integral in creating a shared creative work with another. Because the focus was on the interactions (rather than song quality), the experience was accessible to everyone including those with little or no songwriting experience. One participant noted, “A lot of us are actually first-time songwriters and in fact most of us are first time—some of us aren't even regular musicians so a big part of this program is that anyone can be a songwriter, anyone can be a storyteller.” Another participant described, “I felt like the pressure was off that I didn't have to live up to crazy standards I put myself up to that you all were so open that I was like okay I can do this.”
- *Varied collaboration.* Participants included both students and patients, and they had varied backgrounds in songwriting and music experience. The student participants were in different health professional schools and class years. All participants drew on their experiences to collaborate and share feedback. One participant said, “Being able to bounce ideas off someone else, I was able to get out of my own head a little bit and get something on a paper.” Another participant said, “I brought in some ideas and then a bunch people listen to it and had some pointers in that, that helped me. As soon as I got home after that I wrote more.”

Q2: What was the extent of attitude change in student participants?
  The mean pre- and post-program Jefferson Scale scores of student participants were 5.92 and 6.36, respectively, which was a significant difference (Wilcoxon, z = 2.03, p < 0.05). There was no significant difference in pre- and post-scores of the Interpersonal Reactivity Index (Wilcoxon, z = 1.10, p > 0.05).

## Discussion

In this program, collaborative songwriting is a participatory art involving both patients and students. To explore how an interpersonal approach could work in practice, we were interested in processes underlying interactions and engagements (Q1). Interviews with ten student participants and focus group responses revealed the following processes of interpersonal interactions: reciprocal relationships, interactions in the community, joint goal, and varied collaboration. We were also interested in whether attitudes about care increased pre- and post- program, measured by the Jefferson Scale (Q2). Our prediction that attitudes about care would increase after participation in the program was supported, although the low number of participants completing surveys (n = 7) limits interpretations of the results in this pilot evaluation. We made no prediction about changes in trait empathy as measured by the Interpersonal Reactivity Index, and these results were inconclusive.

Although student and patient participants were included in the program and the focus group discussion, the Jefferson Scale is designed for health professionals, and thus we distributed it to health professional student participants only. Scales of empathy or attitudes about care in medical education reflect the current state of involving students only in programs in the medical humanities. In adopting an interpersonal approach, programs that include both patient and student participants may consider using interpersonal measures not limited to empathy, such as interpersonal closeness, trust, and belonging (e.g. the Inclusion of the Other in the Self Scale, a measure of interpersonal closeness: see Aron, Aron, and Smollan 1992).

The present program is innovative for its interpersonal approach of involving both students and patients in a collaborative creative process. It was a student-organized activity, and students encouraged classmate participation regardless of prior musical experience. Although many participants had prior musical experience, most of the students and patients had never written a song before and the experience of doing so with others was a motivating factor for them to try for the first time. Songwriting assistance was given through a songwriting guide and peer sessions from those with more experience. This student-generated model of involvement aligns with the co-creative ethos of the activity and its activities may provide opportunities for development of other curricular possibilities such as inclusion within established medical humanities curricula or electives.

Engagement in a shared activity towards a joint goal, whether musical or not, has a role in developing interpersonal closeness and socio-cognitive skills through shared intentionality (Tomasello and Carpenter 2007). In this program, the participatory nature of music further involves synchronized activity which tends to promote positive social affiliation (Hove and Risen 2009; Valdesolo and DeSteno 2011). While many forms of participatory arts can build such affiliation such as through collaborative visual or written art, music’s temporality and ambiguity of semantic meaning makes it particularly effective in allowing for social synchrony in situations of social uncertainty (Cross 2014). Future workshops and programs may incorporate other collaborative arts in addition to music, such as also involving visual arts and creative writing.

This program can serve as an example of a starting point towards incorporation of the participatory arts and interpersonal approach towards justice-oriented approaches. However, there were limits towards ideal interpersonal reciprocity. The program could enhance interpersonal inclusion further if students and patients had opportunities to work together on song creation in between the first conversation session and second song sharing session. In this program, these opportunities were limited by logistics of transporting patients outside of support group sessions, but other settings in which patients and students could meet more frequently could allow greater collaborative possibilities. Thus, while not reaching an ideal of co-creation, it was one step in applying a participatory arts approach with patients in an expansion of typical application of the presentational arts. It may be helpful to view an interpersonal approach as existing on a spectrum, with high interpersonal inclusion on one end and low interpersonal inclusion on the other, and the current intervention as falling in the middle. Degree of inclusion of interpersonal elements in a justice-promoting manner may be aided by a framework based on the qualitative themes (Q1), which we describe next.

## Framework for an interpersonal approach

By aligning critical and narrative theory with the themes underlying the interactive nature of the project, we propose a theoretically-informed framework for an interpersonal approach in the medical humanities. The framework may provide a starting place for the application of the participatory arts in clinical and educational contexts.

### Center around reciprocal relationships

Fraser (1995) describes transformative processes as those that “change *everyone's* sense of belonging, affiliation, and self” (81). “Transformative remedies reduce social inequality without, however, creating stigmatized classes of vulnerable people perceived as beneficiaries of special largesse. They tend therefore to promote reciprocity and solidarity in the relations of recognition" (85–86). Despite the inherent power imbalances that exist between relations such as that between physician and patient, interventions that allow for reciprocal and mutual interactions foster cultures of just care towards transformative remedies. Music-making and other participatory arts allow for relationships where the creation process is reciprocally interactional.

### Build interactions in community settings

Medical humanities programs are often held in the classroom setting or in one-off instances. For example, students may read a poem and discuss the poem with each other (Collett and McLachlan 2006; Shapiro and Rucker 2003). These activities usually take place in a classroom rather than in a clinical or community setting. An interpersonal approach encourages relationships to develop with community members in their own settings, where group cultures emerge and change through interactional processes between students and community members. Service-learning is one example of a community-based approach (Hunt 2011). Service-learning models are generative examples of interventions in community settings but need be wary of not falling into non-reciprocal models of interaction that prioritize student learning over joint interaction with community members (e.g., see Eliasoph 2013).

### Have joint goals

Interventions may have an individual goal such as personal reflection through writing or a shared goal such as creating collaborative creative art. In *Pedagogy of the Oppressed* (2018), Paulo Freire describes both teacher and student as learners, as “co-investigators” engaging in a shared task. Through joint aims, the boundaries between the “serving” and “served” can be oriented towards agency for all involved. Shared intention can be even more meaningful when the goal focuses on a social intent rather than on the quality of the work produced (Cao 2013). Forms of theater and improvisation, for example, focus on a democratic process seeking to engage in dialogue and create space for collective action (Boal 1974).

### Value varied collaboration

Collaboration across skills, experiences, and backgrounds are a fundamental part of coalition-building—one goal Fraser identifies in calling for justice-oriented remedies. Participatory arts foster skills-based collaboration given the multifaceted nature of these arts. In collaborative songwriting, for example, components involve lyrics, instrumentals, melody, and production. Each component involves participants to draw on their own experiences or interests to create a complete piece. Likewise, the health professions require interprofessional collaboration and coalition-building to advance optimal health outcomes (D’Amour et al. 2005; Zwarenstein, Goldman, and Reeves 2009).

### Evaluate outcomes based on interpersonal measurements for all participants

Unlike trait measures such as the Interpersonal Reactivity Index of empathy, interpersonal measurements are dependent on interactions between people, such as norms, attitudes, trust, and interpersonal closeness. Other than the Jefferson Scale on attitudes of care in health professionals, measures that may be considered interpersonal include the Inclusion of the Other in the Self Scale and connectedness, trust, and need-to-belong measures (Aron, Aron, and Smollan 1992; T. van Bel et al. 2009; Justwan, Bakker, and Berejikian 2018; Nichols and Webster 2013). Such scales can be adapted and applied to every population group involved in the interactions (e.g., patients and students); for example, the Inclusion of the Other in the Self Scale simply requires measurement of the overlap between two circles labeled “self” and “other,” applicable to members of any group in a relational context.

Rather than approaching empathy as skills or traits from an individual standpoint, these themes of reciprocal relations, community interactions, joint goals, varied collaboration, and interpersonal measurements focus on the social contexts which determine how and in what manner relations are structured and engaged in. Such development of interpersonal empathy ultimately allows for the development of empathic capabilities in individuals as well, advancing an expanded understanding and application of empathy more closely aligned with its historical roots in a nineteenth century sympathy of moral sentiment and social norms.

## Conclusion

The medical humanities can broaden perspectives and deepen appreciation; they can cultivate skills of empathy, listening, and observation in health professionals. But they can and should do more and look critically at empathy or individual skill-based approaches. We have proposed an interpersonal approach of incorporating the arts and humanities in ways to go beyond the development of individual empathy in health professionals—for instance, through the participatory arts which have been relatively neglected in the curricula but have much to offer. The ideas here are not intended to be an “either/or” pronouncement but a “yes, and” possibility that the medical humanities shift and expand, and that this expansion is not only important for the medical humanities as a field but aligned with justice-oriented aims in medicine and health care (such as with public health and the health humanities). We want students to learn through the arts and humanities, not only so that they may possess or develop special powers of empathy or understanding but also so that they have the shared responsibility of improving the quality and equity of health as co-creators among their peers, colleagues, humanists, patients, and the public.

## Appendix A: Song Ideas Form

1. Share about yourselves.
  - Favorite places:
  - Favorite quotes:
  - Favorite words:
  - People special to you:
2. What kind of music do you both like? What genre (e.g., pop, rock, rap, classical, jazz)?
3. Do you share any favorite songs or artists?
4. What is an experience or story important to you? You could each share a story! (This could be what the song is about. Some examples: a dream or goal that you have, a certain person who’s special to you and why, a good memory or experience, etc.)
5. What is something you both believe in or a message you’d like to share with others? (This could be the main message of the song.)
6. What are specific words or phrases that you both like? (These could be included in the song.)
7. What else would you like to share about yourselves?

## Appendix B: Interview Questions Guide

1. Tell me about your experience of writing a song.
2. How did you interpret story/experiences and reimagine it in song?
3. What was the most meaningful aspect of the project?
4. Do you feel like you understood others better through this project? How so?
5. What was it like to share the song?
6. What aspects of the program could be improved upon?
